# The Potential Use of Radiomics with Pre-Radiation Therapy MR Imaging in Predicting Risk of Pseudoprogression in Glioblastoma Patients

**DOI:** 10.3390/jimaging7020017

**Published:** 2021-01-28

**Authors:** Michael Baine, Justin Burr, Qian Du, Chi Zhang, Xiaoying Liang, Luke Krajewski, Laura Zima, Gerard Rux, Chi Zhang, Dandan Zheng

**Affiliations:** 1 Department of Radiation Oncology, University of Nebraska Medical Center, Omaha, NE 68198, USA; mbaine@unmc.edu (M.B.); justin.burr@unmc.edu (J.B.); lkrajewski@unmc.edu (L.K.); laura.zima@unmc.edu (L.Z.); GerardRux@creighton.edu (G.R.); chi.zhang@unmc.edu (C.Z.); 2 Department of Biological Science, University of Nebraska Lincoln, Lincoln, NE 68588, USA; qian.du@huskers.unl.edu (Q.D.); zhang.chi@unl.edu (C.Z.); 3 Department of Radiation Oncology, University of Florida Proton Institute, Jacksonville, FL 32206, USA; XLiang@floridaproton.org

**Keywords:** radiomics, glioblastoma, GBM, pseudoprogression, radiation

## Abstract

Glioblastoma (GBM) is the most common adult glioma. Differentiating post-treatment effects such as pseudoprogression from true progression is paramount for treatment. Radiomics has been shown to predict overall survival and MGMT (methylguanine-DNA methyltransferase) promoter status in those with GBM. A potential application of radiomics is predicting pseudoprogression on pre-radiotherapy (RT) scans for patients with GBM. A retrospective review was performed with radiomic data analyzed using pre-RT MRI scans. Pseudoprogression was defined as post-treatment findings on imaging that resolved with steroids or spontaneously on subsequent imaging. Of the 72 patients identified for the study, 35 were able to be assessed for pseudoprogression, and 8 (22.9%) had pseudoprogression. A total of 841 radiomic features were examined along with clinical features. Receiver operating characteristic (ROC) analyses were performed to determine the AUC (area under ROC curve) of models of clinical features, radiomic features, and combining clinical and radiomic features. Two radiomic features were identified to be the optimal model combination. The ROC analysis found that the predictive ability of this combination was higher than using clinical features alone (mean AUC: 0.82 vs. 0.62). Additionally, combining the radiomic features with clinical factors did not improve predictive ability. Our results indicate that radiomics is potentially capable of predicting future development of pseudoprogression in patients with GBM using pre-RT MRIs.

## 1. Introduction

Imaging is a primary diagnostic tool upon which medical decisions are made, and magnetic resonance imaging (MRI) is commonly used to monitor post-treatment effects for central nervous system (CNS) tumors. There is more information within images than is initially seen, however. Radiomics is the transformation of images to mineable data [1]. The process of radiomics involves image acquisition, segmentation, and labeling of the tumor/normal tissues, extraction of quantitative features (shape, intensity, texture), followed by statistical modeling and machine learning [2]. Radiomics is of interest in oncology, as it has the potential to provide important diagnostic and prognostic information. Cellular and molecular changes impacting imaging characteristics may allow for the gathering of complex tumor information through less invasive methods. Furthermore, these features are readily available as patients have many images taken throughout the course of their treatment.

One category of tumor that could benefit from the clinical applications of radiomics is gliomas. Gliomas are the most common primary CNS malignancy in adults and are separated into grades I-IV according to the World Health Organization (WHO) [3]. Glioblastoma (GBM), WHO Grade IV, are the most lethal and most common gliomas in adults, with more than 10,000 cases diagnosed per year in the United States [4]. GBMs grow rapidly and are extremely invasive. Treatment for GBM typically involves maximal safe resection with adjuvant chemoradiation (CRT). Even with aggressive treatment of radical surgical resection and adjuvant CRT, the median survival is only 15–20 months [5,6]. Known prognostic factors for patients with GBM include patient age, performance status, tumor grade and histology, MGMT promoter methylation IDH-1 mutation, and the extent of resection [3,7]. As the disease progression varies among patients and can be rapid, close monitoring, especially with medical imaging, is important.

Following completion of treatment, imaging with brain MRIs is scheduled per NCCN guidelines. Pseudoprogression is a transient radiologic finding frequently encountered in the post-radiotherapy (RT) setting that often imitates true progression, with or without clinical effects [8]. Varying in incidence from 12%–50%, pseudoprogression is associated with an increased overall survival in GBM patients, but differentiating this entity from true progression presents a difficult task for oncologists seeking to give effective treatment [9,10,11].

Developing pseudoprogression is partially due to inherent features of tumors. For example, pseudoprogression is more commonly seen in patients with MGMT promoter methylation and IDH-1 mutations [10,11,12,13]. Tumors with MGMT promoter methylation exhibit pseudoprogression in up to 91% of cases versus 41% of cases in those unmethylated [12]. Similarly, pseudoprogression was seen in 54.1% of patients with IDH-1 mutations [11]. However, pseudoprogression occurs in patients without these molecular features, highlighting the need for additional tools to predict pseudoprogression or distinguish pseudoprogression from true progression.

Most efforts attempting to distinguish pseudoprogression from true progression on follow-up imaging utilize known parameters, including tumor size, edema changes from T1- and T2-weighted sequences, and diffusion and perfusion indices. Collectively, these parameters have proven insufficient to reliably discriminate pseudoprogression from true progression [14]. Thus, radiomic methods have been explored to address these challenges. As a first step in the investigation of using complex imaging information, Jang et al., utilized raw MRI images and deep learning methods for predicting pseudoprogression vs. true progression with moderate predictability achieved, with a mean AUC (area under ROC (receiver operating characteristic) curve) at 0.72 in the training set. While novel in the field, the results indicate the necessity of further studies [15]. To our knowledge, no study has utilized radiomics for predicting pseudoprogression using pre-RT scans, which may provide information on intrinsic tissue factors of patients prone to develop pseudoprogression.

The clinical potential of radiomics has not been reached, and the amount of clinically relevant data in the field is sparse [16]. Our goal is to assess the clinical utility of radiomics for patients with GBM by extracting radiomic features predictive of pseudoprogression. Our hypothesis is that radiomic features extracted from pre-RT imaging will be able to suggest pseudoprogression.

## 2. Materials and Methods

### 2.1. Patient Information and Clinical Data

This study was approved by the institutional review board (Protocole#398-17-EP, approved 26 June 2017). Our database included patients with pathologically proven glioblastoma between 2009 and 2018. Patients with GBM who had pre-RT MRIs were included. Of the 72 patients fitting these parameters, 35 reached final analysis due to others not having pseudoprogression data. Data were gathered via a retrospective chart review in a single department at a Midwest medical school, and they included demographics, pathologic characteristics, treatment, recurrence history, and pre-RT MRI images. Clinical features included age, gender, location of tumor, and extent of resection. These features were selected independent of and in combination with radiomic features to evaluate their utility in predicting pseudoprogression. Radiomic data were extracted exclusively from pre-RT imaging and analyzed to determine pseudoprogression prediction. Pseudoprogression was determined by reviewing post-RT imaging and clinical notes. Pseudoprogression was defined as post-treatment findings on imaging that either resolved spontaneously or with steroids on subsequent imaging.

### 2.2. Imaging, Segmentation, and Feature Extraction

#### 2.2.1. Imaging

Pre-RT T1 MRI images with gadolinium-based contrast agents were taken before radiation therapy. These contrast-enhanced T1 MRI images were used for radiomic analysis. The radiomic features analyzed were from pre-RT scans only. Pre-treatment imaging was chosen for this analysis due to the concept that development of pseudoprogression is likely multi-factorial but partially due to tumor features such as the amount/nature of new vasculature, growth rate, association with tumor stroma, and the genetic profiles of the malignancies. The ability to extract and analyze these differences through a radiomic analysis may be compromised by post-treatment effects in the tumor. Sequential images (usually every 3 months) were acquired following RT. These post-radiation MRI images were used to assess pseudoprogression. Only patients with regular follow-up and sufficient numbers of post-radiation MRIs for pseudoprogression assessment were included in the final analysis. Pseudoprogression was diagnosed from a consensus of the CNS oncology treatment team, which includes certified neuroradiologists, radiation oncologists, neurosurgeons, and medical oncologists. All MRI images were acquired on a 1.5 T Ingenia MRI scanner by Philips Medical Systems. Slice thickness ranged from 1 to 2.5 mm, with most using 1 mm slices. 

#### 2.2.2. Segmentation

Regions of interest, manually drawn by one of two attending radiation oncologists according to the tumor boundary on the contrast-enhanced T1-weighted pre-RT MRIs, were used for analysis. Segmentation based on contrast-enhanced T1 imaging without use of T2 flair imaging was chosen for multiple reasons: (1) consistency in segmentation across patients and amongst the radiation oncologists providing the segmentation; (2) pseudoprogression often occurs in the treated regions receiving full radiation dose, which is a volume based on the contrast-enhanced T1 imaging regardless of which guidelines the respective radiation oncologist used for treatment planning; and (3) the flair portion of an MRI in the setting of glioblastoma is of unclear etiology, potentially representing tumor infiltration, benign edema, or recent seizure activity. Our intention was to analyze radiomic features based on characteristics of the tumors, focusing on the area of greatest likelihood to be attributable to the malignancy (contrast-enhancing region). To avoid bias field distortions and data heterogeneity bias, a bias field correction using N4 and an image normalization using histogram matching was performed using the 3D Slicer software on the MRI images before feature extraction.

#### 2.2.3. Radiomic Feature Extraction

From each segmented tumor, 841 radiomic features were extracted from the segmented 3D volume using the radiomics module on 3D Slicer 4.9 [17] and visualized using an interactive visualization platform [18]. A resampled 3 × 3 × 3 mm^3^ voxel size and a bin width of 25 were used for feature extraction. The features are defined in compliance with feature definitions as described by the Imaging Biomarker Standardization Initiative (IBSI) [19] and can be divided into original features (105 features) and wavelet features (736 features). The original features can be sub-divided into 7 classes, including 13 shape features, 18 first order statistical features, 23 gray level co-occurrence matrix (GLCM) features, 14 gray level dependence matrix (GLDM) features, 16 gray level run length matrix (GLRLM) features, 16 gray level size zone matrix (GLSZM) features, and 5 neighboring gray tone difference matrix (NGTDM) features. The wavelet features included all except shape features calculated on the filtered images with all 8 combinations of applying either a high or a low pass filter in each of the three dimensions. For our experiment, a 16GB RAM system with an Intel Core i7-7700 CPU processor @3.60 GHz was used. The feature extraction took on average 2–3 min per patient image set. A list of all features are given in Supplementary Table S1.

### 2.3. Data Analysis

The data analysis, described with more details in the following sections, was performed by using R (version 3.3.2) [20].

#### 2.3.1. Heatmap Analysis of Variables

To investigate the overall relationship of radiomic features, a heatmap analysis was performed. Hierarchical clustering was conducted with the average method based on the correlation distance for both radiomic features and patients. Patients were grouped into two clusters, and each cluster has a unique pattern.

#### 2.3.2. Radiomic Feature Selection

For the radiomic prediction, feature selection was performed with the following steps. A univariate ANOVA analysis was performed to select potential features contributing to the classification. In the ANOVA analysis, patients with pseudoprogression and those without were assumed to have equal means for the tested radiomic feature, indicating that the feature provides little information for differentiating the two groups. A significant *p*-value rejects the assumption, which means the tested feature can provide information for the prediction of pseudoprogression. Since hundreds of radiomic features were tested independently, false discovery rates (FDRs) were calculated to reduce the impact of the multiple testing problem. Those features with FDR-adjusted *p*-values smaller than 0.1 were selected. They were then clustered to group highly correlated features (cutoff = 0.9) into clusters, and only the feature with the lowest FDR adjusted *p*-value was kept within each cluster to reduce collinearity. Lastly, sequential floating forward selection (SFFS) was then used to select features, which gave the highest area under the receiver operating characteristic (ROC) curve (AUC) value with a random forest machine learning model. The PRAUC values were also calculated from the precision-recall ROC curves.

#### 2.3.3. Radiomic Model Building and Validation

The prediction model was trained with random forest to optimize the AUC. The performance of the model was evaluated by 1000-time 3-fold cross-validations, yielding 3000 AUC values from the test datasets. The statistical distributions of these 3000 AUC values were used to describe the performance of the model. Due to the uneven patient distribution between the pseudoprogression and non-pseudoprogression groups (8:27), a stratified 3-fold cross-validation was used, i.e., the ratio of positives to negatives of the data in each group was restricted in order to have the same ratio as in the whole dataset. Therefore, subsets are representative of all strata of the whole dataset. Three top-ranked radiomic features from feature selection were used to generate 1-feature, 2-feature, and 3-feature radiomic models, yielding a total of 7 different models. The model with the highest average AUC value was selected as the final radiomic model. A one-tailed t-test was conducted to compare each of the other models with this model, with a null assumption that the other comparing model is better than the selected model. 

#### 2.3.4. Radiomic and Clinical Model Comparison

The model performance of the following three signatures were evaluated: the radiomic signature (with the selected radiomic features as described in Section 2.3.3), the clinical signature (with the selected clinical features as described in Section 2.1), and the combined signature (with both the selected radiomic features and clinical features). The clinical features included age, gender, location of tumor, and extent of resection. For this comparison, a ROC analysis was performed with an additional 1000-round stratified 3-fold cross-validations on the above three signatures, from which the AUC, the PRAUC, the true positive rate (TPR), and the true negative rate (TNR) of the trained models were calculated on the test datasets and compared amongst the three models. 

## 3. Results

A total of 72 patients were identified for this study, of whom 35 patients could be assessed for pseudoprogression and were thus included in the analysis. The patient, treatment, and tumor characteristics are reported in Table 1. There were 24 patients (68.6%) who were males and 11 (31.4%) were females. The mean age was 55.5 with a range of 8– 87 years old. MGMT promoter and IDH1 statuses of the cohort were largely unknown, as seen in Table 1. Almost 75% of MGMT promoter and 50% of IDH1 statuses were unknown, making the positive or negative identification of pseudoprogression more difficult. Most GBM patients in our study were treated with 60 Gy in 30 fractions (21/35, 60%), with other regimens being less common. Similarly, concurrent temozolomide (TMZ) was given to 71.4% (25/35) of patients and 74.3% (26/35) of patients were treated with adjuvant TMZ. Pseudoprogression was found in 8 (22.9%) cases.

**Figure 1:**
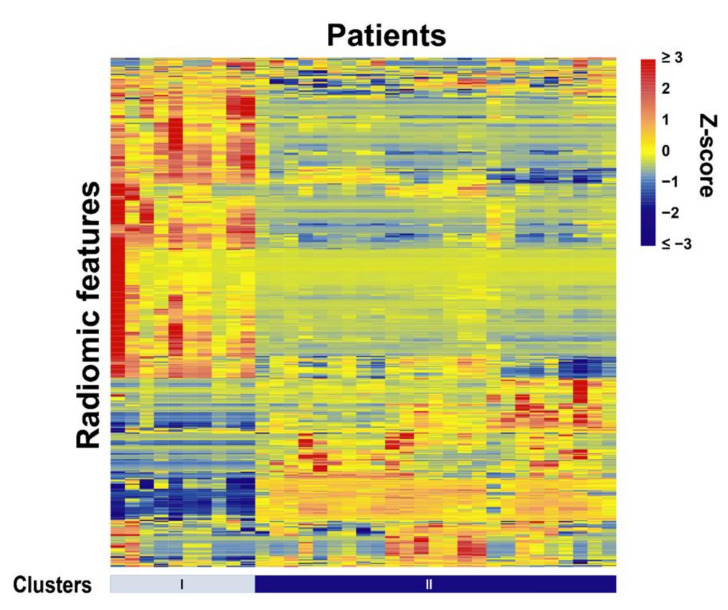
Radiomic heat map shows unsupervised clustering of all patients. The patients were clustered into 2 clusters based on the similarity of their radiomic feature patterns. The standardized z-scores were used to depict the normalized feature value variation for the patients.

A heatmap of all the radiomic features and patients is plotted in Figure 1. Using the cross-validations, from the 841 radiomic features, three top-ranked features were selected. As given in Table 2, the selected features included wavelet_HHL_firstorder_Mean (feature 1), original_firstorder_Minimum (feature 2), and wavelet_LHL_glszm_SizeZoneNonUniformityNormalized (feature 3). Feature 2 is derived from the original image, representing the minimum intensity within the volume of interest. Features 1 and 3 are derived from wavelet filtered images with high-pass, high-pass, and low-pass filters, and low-pass, high-pass, and low-pass filters, along the x, y, z directions, respectively. Feature 1 represents the average intensity within the volume of interest on the derived image. Feature 3 represents the normalized variability of size zone volumes throughout the volume of interest where a lower value indicates more homogeneity. The AUCs (average and standard deviation) from the 1000-time 3-fold cross-validation are also listed in Table 2 for models using these features (univariate prediction).

Among the 7 radiomic models (3 single-feature models, 3 two-feature models, and 1 three-feature model), the single-feature models yielded mean AUCs between 0.6 and 0.7, while the two-feature and three-feature models yielded AUCs above 0.7. The two-feature model combining features 2 and 3 achieved the highest mean AUC of 0.82. *P*-values were above 0.05 for all *t*-tests comparing the other 6 models with this model, which leads us to reject the hypotheses that any other model is superior to this model. The AUCs, PRAUCs, F1 scores from the precision-recall curves, TPRs, and TNRs from the two-feature and three-feature models are listed in Table 3. For the selected model with features 2 and 3, a cutoff logit value of −0.754 would optimally yield a true positive rate of 0.8 while maintaining a false positive rate around 0.25. In application, patients with calculated logit values above the cutoff value are predicted to be prone to developing pseudoprogression following radiotherapy.

**Figure 2:**
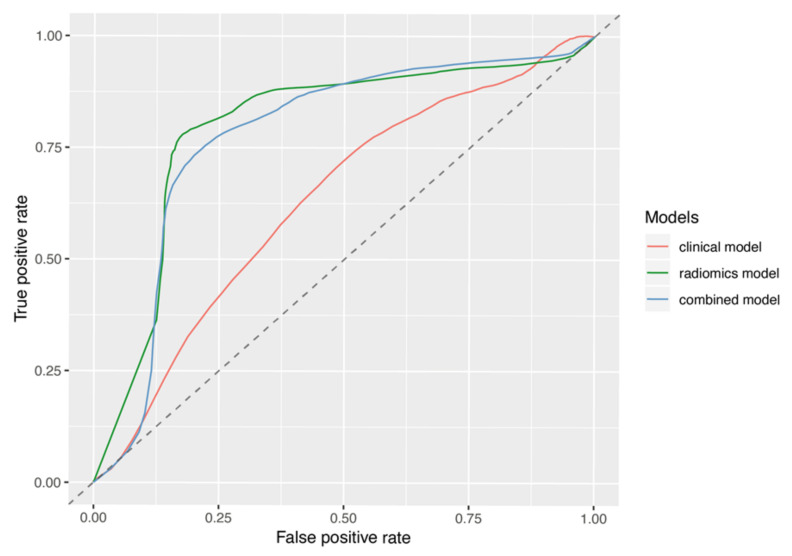
The average ROC curves of the selected radiomics model, the clinical model, and the combined model with both radiomic and clinical features, generated from the 3000 cross-validation datasets. The mean AUC was 0.62, 0.82, and 0.80 for the clinical model, the radiomics model, and the combined model, respectively.

**Figure 3:**
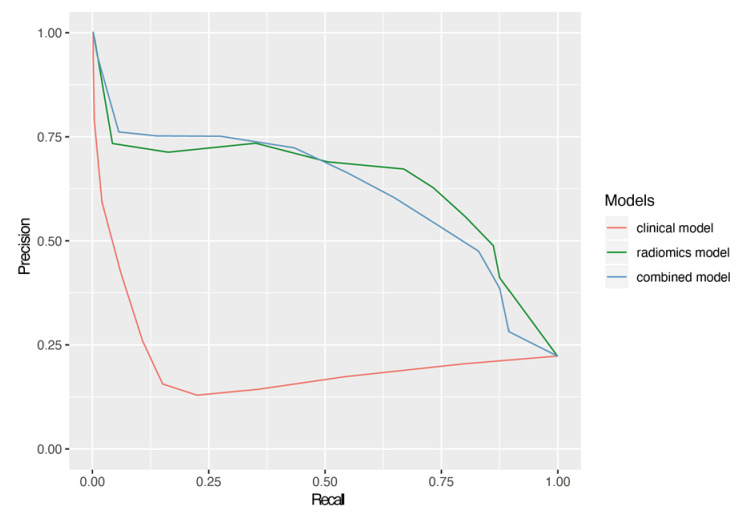
The average precision-recall ROC curves of the selected radiomics model, the clinical model, and the combined model with both radiomic and clinical features. The mean PRAUC was 0.21, 0.62, and 0.62 for the clinical model, the radiomics model, and the combined model, respectively.

The ROC analysis comparing the clinical-based model, the radiomics-based model, and the combined model yielded mean AUCs of 0.62, 0.82, and 0.80 from the 1000 rounds of stratified 3-fold cross-validations, respectively; this suggests that the radiomic signature was superior to the clinical signature and is similar to the combined model at predicting the development of pseudoprogression. A comparison of the average ROC curves and precision-recall ROC curves of the three models is shown in Figure 2 and Figure 3, respectively. As shown in both figures, the radiomics and combined models are comparable while the clinical model shows a significant decrease in efficacy. Table 4 also lists the details of the AUCs, PRAUCs, F1 scores from the precision-recall curves, TPRs, and TNRs for the comparison.

## 4. Discussion

In this study, our objective was to identify radiomic features to predict pseudoprogression in GBM patients using pre-RT MRI imaging as a pilot feasibility study. A radiomic model was constructed using 1000 times 3-fold cross-validations, which achieved a mean AUC of 0.82 on the 3000 test datasets with a random forest model using a combination of two selected features. These two radiomic features were then compared with four clinical features and a model including both radiomic and clinical features through ROC analyses with an additional 1000-round 3-fold cross-validations. The radiomic model resulted in a mean AUC of 0.82, which was higher than the clinical model (0.62). Combining radiomic and clinical features did not improve the model performance, resulting in a mean AUC of 0.80. These results demonstrate the potential of radiomics in predicting pseudoprogression using pre-RT MRIs.

These results fit within the context of previously published works using advanced imaging analyses, including radiomics and radiogenomics in differentiating pseudoprogression from true progression [15,21,22,23]. Several studies have also aimed to predict overall survival, progression free survival, EGFRvIII and IDH1 mutation status, and MGMT promoter methylation status using radiomics [24,25,26,27]. Li et al., successfully developed a multi-parametric signature using preoperative imaging that predicted overall survival in patients with GBM more accurately than conventional prognostic factors or a fixed-parameter radiomics model [24]. The combination of a radiomics model with clinical and genetic profiles improved the predictability for overall and progression-free survivals [26]. Xi et al. added to the existing body of literature by suggesting that radiomic features could predict MGMT methylation status in GBM pre-operatively [25]. Furthermore, Soike et al. found that true imaging response was associated with an improved overall and progression-free survival, and that MGMT methylation correlated with true imaging response but not pseudoprogression [27].

Our study investigated the ability of radiomics to predict pseudoprogression. Pseudoprogression remains an important phenomenon in GBM as its differentiation from true treatment failure is imperative for patient management. Pseudoprogression can often be managed with observation alone, while true progression requires therapeutic alteration or consideration of discontinuation of care [8,28]. A previous study from Jang et al. showed promising results, as they studied radiomics in post-chemoradiation therapy (post-CRT) MRI scans with moderate predictability [15]. One concern raised from studying post-CRT MRI scans was the potential distortion of intrinsic radiomic features in the brain due to treatment effects. Using pre-RT imaging, we found a combination of two radiomic features predictive of development of future pseudoprogression. These radiomic features showed an increase in the predictive ability when compared to clinical features and dominated the performance in the combined model. The two selected features are the minimum value within the region-of-interest on the original image and SizeZoneNonUniformityNormalized on the derived image after applying a low-pass, a high-pass, and a low-pass wavelet filter along the x, y, z directions, respectively. SizeZoneNonUniformityNormalized is a normalized value that measures the variability of size-zone volumes throughout the region of interest, with a lower value indicating more homogeneity among zone-size volumes in the image.

Limitations of our study include the relative unknown nature of molecular sub-types within our population, including MGMT and IDH-1 statuses, which have been shown to be linked to a higher incidence of pseudoprogression and thus may confound our data [11,12]. Due to the incomplete marker status in our retrospective cohort, such factors were not included in our study to avoid further reducing the analyzable data size. Future studies are warranted to analyze such factors and evaluate their associations with the radiomic features. In addition, the region of interest segmentation was performed by two physicians, but the inter- and intra-observer variability was not studied. Although it has been shown that pseudoprogression may be related to an increased survival time, survival data were not collected as this has already been reported in previous studies [29,30]. Furthermore, the retrospective nature, limited number of patients, relative homogeneity of the studied population, and inclusion of patients from a single institution may limit the ability of this data to be extrapolated to other populations. The post-surgical treatment modalities the studied patients received were heterogeneous with RT dosing varying from hypofractionated palliative doses to a standard conventionally fractionated dose, which also could confound the results. The limited sample size of the final analyzable patient population and the lack of an independent validation group are major limitations of the current work. These limitations may impact the uncertainty and generalizability of our findings. Nevertheless, efforts were made to best address some of these limitations, such as performing 1000-round 3-fold cross-validations in feature selection to mitigate the limited sample size. In addition, only 2 features were used in the final radiomic model in an uncertainty attempt to minimize the chance of model overfitting.

Despite these limitations, results from current studies support the potential clinical applications of radiomics to predict pseudoprogression. As patients are followed closely with imaging, the value in differentiating changes on these scans with reliable predictability would be immense.

Future directions of this work include larger scaled studies with a validating database to confirm these results. In addition, expanding radiomic analyses to post-RT scans, and combining pre-RT and post-RT radiomic features, such that intrinsic tissue factors and post-treatment changes can be incorporated into predictive algorithms to identify pseudoprogression, could prove worthwhile. Furthermore, studies involving molecular marker status to control for these variables would be valuable as well.

## 5. Conclusions

Glioblastoma is the most common glioma in adults, and pseudoprogression is a well-defined post-treatment effect frequently encountered. Using radiomic data from pre-radiation therapy MRI images, an optimal signature of two radiomic features out-performed clinical features alone in predicting the development of future pseudoprogression. Additionally, when combining radiomic features with clinical features, performance did not improve. Further large-scale studies are needed to validate our findings, but the results indicate the potential of radiomic features to predict future development of pseudoprogression in GBM patients using pre-radiation therapy MRIs. 

## Figures and Tables

**Table 1:** Patient demographics of the study population. Clinical features of chemotherapy, radiation dose, location, and extent of resection are included as well. MGMT: O6-methyl-guanine methyl transferase; IDH: isocitrate dehydrogenase; TMZ: temozolomide; Gy: Gray; Fx: fractions; GTR: gross total resection; NTR: near-total resection; STR; sub-total resection.

	Total (*n* = 35)		Pseudoprogression (*n* = 8)		No Pseudoprogression (*n* = 27)	
Age	55.5 (8–87)		59.3 (26–73)		54.3 (8–87)	
Gender	*n*	%	*n*	%	*n*	%
Male	24	68.6	7	87.5	17	63.0
Female	11	31.4	1	12.5	10	37.0
MGMT Promoter status						
Methylated	7	20	2	25	5	18.5
Non-methylated	3	8.6	1	12.5	2	7.4
Unknown	25	71.4	5	62.5	20	74.1
IDH-1 status						
Wild-Type	13	37.1	3	37.5	10	37.0
Mutant	4	11.4	1	12.5	3	11.1
Unknown	18	51.4	4	50	14	51.9
Concurrent TMZ						
Yes	25	71.4	6	75	19	70.4
No	8	22.9	2	25	6	22.2
Unknown	2	5.7	0	0	2	7.4
Adjuvant TMZ						
Yes	26	74.3	5	62.5	21	77.8
No	6	17.1	2	25	4	14.8
Unknown	3	8.6	1	12.5	2	7.4
Radiation Dose/Fractions						
60Gy/30Fx	21	60	5	62.5	16	59.3
59.4Gy/33Fx	4	11.4	0	0	4	14.8
54Gy/30Fx	2	5.7	0	0	2	7.4
55.8Gy/31Fx	1	2.85	1	12.5	0	0
40.05Gy/15Fx	2	5.7	0	0	2	7.4
34.6Gy/11Fx	1	2.85	0	0	1	3.7
34Gy/10Fx	2	5.7	1	12.5	1	3.7
25Gy/5Fx	1	2.85	1	12.5	0	0
Unknown	1	2.85	0	0	1	3.7
Location						
Right frontal lobe	8	22.9	2	25	6	22.2
Right temporal lobe	6	17.1	0	0	6	22.2
Right parietal lobe	4	11.4	1	12.5	3	11.1
Left frontal lobe	0	0	0	0	0	0
Left temporal lobe	3	8.6	1	12.5	2	7.4
Left parietal lobe	5	14.2	1	12.5	4	14.8
Other location	3	8.6	2	25	1	3.7
Unknown	6	17.1	1	12.5	5	18.5
Extent of resection						
GTR	12	34.2	1	12.5	11	40.7
NTR	3	8.6	1	12.5	2	7.4
STR	11	31.4	2	25	9	33.3
Biopsy	7	20	4	50	3	11.1
None	1	2.85	0	0	1	3.7
Unknown	1	2.85	0	0	1	3.7

**Table 2:** Three radiomics features of 841 selected from each segmented tumor. Univariate AUCs and PRAUCs for the three radiomics features are listed. AUC: area under ROC curve; Std. Dv.: standard deviation; PRAUC: area under the precision-recall ROC curve.

Radiomic Feature ID	Radiomic Feature	Mean AUC	Std. Dv.	Mean PRAUC	Mean F1
1	wavelet_HHL_firstorder_Mean	0.66	0.19	0.51	0.52
2	original_firstorder_Minimum	0.67	0.18	0.47	0.37
3	wavelet_LHL_glszm_SizeZoneNonUniformityNormalized	0.66	0.20	0.53	0.47

**Table 3:** ROC analysis results for testing the combination of any two or all three top radiomic features selected from 3000 (1000 repeats of 3-fold cross-validations) tests. The best-performing model based on the mean AUC value was the combination of features 2 and 3. AUC: Area under ROC curve; Std. Dv.: standard deviation; PRAUC: area under the precision-recall ROC curve; TPR: true positive rate; TNR: true negative rate.

Radiomic Feature Combination	1,2	1,3	2,3	1,2,3
Mean AUC	0.80	0.75	0.82	0.81
Std. Dv.	0.14	0.20	0.15	0.15
Mean PRAUC	0.60	0.66	0.62	0.63
Std. Dv.	0.22	0.24	0.26	0.24
Mean F1	0.50	0.50	0.59	0.57
Std. Dv.	0.22	0.25	0.29	0.24
Mean TPR	0.58	0.53	0.64	0.63
Std. Dv.	0.29	0.29	0.35	0.29
Mean TNR	0.82	0.84	0.88	0.85
Std. Dv.	0.14	0.13	0.13	0.13

**Table 4:** ROC analysis results for comparing the selected radiomics model, the clinical model, and the combined model with both radiomic and clinical features on 1000-round stratified 3-fold cross-validations. AUC: Area under ROC curve; Std. Dv.: standard deviation; PRAUC: area under the precision-recall ROC curve; TPR: true positive rate; TNR: true negative rate.

	Clinical Model	Radiomics Model	Combined Model
Mean AUC	0.62	0.82	0.80
Std. Dv.	0.16	0.15	0.16
Mean PRAUC	0.21	0.62	0.62
Std. Dv.	0.11	0.26	0.25
Mean F1	0.09	0.59	0.49
Std. Dv.	0.16	0.30	0.29
Mean TPR	0.09	0.64	0.49
Std. Dv.	0.16	0.35	0.33
Mean TNR	0.83	0.88	0.90
Std. Dv.	0.13	0.13	0.11

## Data Availability

The data presented in this study are available on request from the corresponding author.

## Supplementary Materials

The following are available online at https://www.mdpi.com/2313-433X/7/2/17/s1, Table S1: Radiomic features analyzed in study.
