# Supplementary material for: The Potential Use of Radiomics with Pre-Radiation Therapy MR Imaging in Predicting Risk of Pseudoprogression in Glioblastoma Patients
Source: J Imaging. 2021 Jan 28;7(2):17. doi: 10.3390/jimaging7020017 (PMC8321255; doi:10.3390/jimaging7020017)
Supplement: Supplementary file 1 [file jimaging-07-00017-s001.pdf]

Supplementary Table 1: Radiomics features analyzed in the study.

Image type	Feature class	Feature name
original	shape	Maximum3DDiameter
original	shape	Maximum2DDiameterSlice
original	shape	Sphericity
original	shape	MinorAxis
original	shape	Elongation
original	shape	SurfaceVolumeRatio
original	shape	Volume
original	shape	MajorAxis
original	shape	SurfaceArea
original	shape	Flatness
original	shape	LeastAxis
original	shape	Maximum2DDiameterColumn
original	shape	Maximum2DDiameterRow
original	gldm	GrayLevelVariance
original	gldm	HighGrayLevelEmphasis
original	gldm	DependenceEntropy
original	gldm	DependenceNonUniformity
original	gldm	GrayLevelNonUniformity
original	gldm	SmallDependenceEmphasis
original	gldm	SmallDependenceHighGrayLevelEmphasis
original	gldm	DependenceNonUniformityNormalized
original	gldm	LargeDependenceEmphasis
original	gldm	LargeDependenceLowGrayLevelEmphasis
original	gldm	DependenceVariance
original	gldm	LargeDependenceHighGrayLevelEmphasis
original	gldm	SmallDependenceLowGrayLevelEmphasis
original	gldm	LowGrayLevelEmphasis
original	glcm	JointAverage
original	glcm	SumAverage
original	glcm	JointEntropy
original	glcm	ClusterShade
original	glcm	MaximumProbability
original	glcm	Idmn
original	glcm	JointEnergy
original	glcm	Contrast
original	glcm	DifferenceEntropy
original	glcm	InverseVariance
original	glcm	DifferenceVariance
original	glcm	Idn
original	glcm	Idm
original	glcm	Correlation
original	glcm	Autocorrelation
original	glcm	SumEntropy
original	glcm	SumSquares
original	glcm	ClusterProminence
original	glcm	Imc2

original	glcm	Imc1
original	glcm	DifferenceAverage
original	glcm	Id
original	glcm	ClusterTendency
original	firstorder	InterquartileRange
original	firstorder	Skewness
original	firstorder	Uniformity
original	firstorder	Median
original	firstorder	Energy
original	firstorder	RobustMeanAbsoluteDeviation
original	firstorder	MeanAbsoluteDeviation
original	firstorder	TotalEnergy
original	firstorder	Maximum
original	firstorder	RootMeanSquared
original	firstorder	90Percentile
original	firstorder	Minimum
original	firstorder	Entropy
original	firstorder	Range
original	firstorder	Variance
original	firstorder	10Percentile
original	firstorder	Kurtosis
original	firstorder	Mean
original	glrlm	ShortRunLowGrayLevelEmphasis
original	glrlm	GrayLevelVariance
original	glrlm	LowGrayLevelRunEmphasis
original	glrlm	GrayLevelNonUniformityNormalized
original	glrlm	RunVariance
original	glrlm	GrayLevelNonUniformity
original	glrlm	LongRunEmphasis
original	glrlm	ShortRunHighGrayLevelEmphasis
original	glrlm	RunLengthNonUniformity
original	glrlm	ShortRunEmphasis
original	glrlm	LongRunHighGrayLevelEmphasis
original	glrlm	RunPercentage
original	glrlm	LongRunLowGrayLevelEmphasis
original	glrlm	RunEntropy
original	glrlm	HighGrayLevelRunEmphasis
original	glrlm	RunLengthNonUniformityNormalized
original	glszm	GrayLevelVariance
original	glszm	ZoneVariance
original	glszm	GrayLevelNonUniformityNormalized
original	glszm	SizeZoneNonUniformityNormalized
original	glszm	SizeZoneNonUniformity
original	glszm	GrayLevelNonUniformity
original	glszm	LargeAreaEmphasis
original	glszm	SmallAreaHighGrayLevelEmphasis
original	glszm	ZonePercentage

original	glszm	LargeAreaLowGrayLevelEmphasis
original	glszm	LargeAreaHighGrayLevelEmphasis
original	glszm	HighGrayLevelZoneEmphasis
original	glszm	SmallAreaEmphasis
original	glszm	LowGrayLevelZoneEmphasis
original	glszm	ZoneEntropy
original	glszm	SmallAreaLowGrayLevelEmphasis
original	ngtdm	Coarseness
original	ngtdm	Complexity
original	ngtdm	Strength
original	ngtdm	Contrast
original	ngtdm	Busyness
wavelet-HHH	gldm	GrayLevelVariance
wavelet-HHH	gldm	HighGrayLevelEmphasis
wavelet-HHH	gldm	DependenceEntropy
wavelet-HHH	gldm	DependenceNonUniformity
wavelet-HHH	gldm	GrayLevelNonUniformity
wavelet-HHH	gldm	SmallDependenceEmphasis
wavelet-HHH	gldm	SmallDependenceHighGrayLevelEmphasis
wavelet-HHH	gldm	DependenceNonUniformityNormalized
wavelet-HHH	gldm	LargeDependenceEmphasis
wavelet-HHH	gldm	LargeDependenceLowGrayLevelEmphasis
wavelet-HHH	gldm	DependenceVariance
wavelet-HHH	gldm	LargeDependenceHighGrayLevelEmphasis
wavelet-HHH	gldm	SmallDependenceLowGrayLevelEmphasis
wavelet-HHH	gldm	LowGrayLevelEmphasis
wavelet-HHH	glcm	JointAverage
wavelet-HHH	glcm	SumAverage
wavelet-HHH	glcm	JointEntropy
wavelet-HHH	glcm	ClusterShade
wavelet-HHH	glcm	MaximumProbability
wavelet-HHH	glcm	Idmn
wavelet-HHH	glcm	JointEnergy
wavelet-HHH	glcm	Contrast
wavelet-HHH	glcm	DifferenceEntropy
wavelet-HHH	glcm	InverseVariance
wavelet-HHH	glcm	DifferenceVariance
wavelet-HHH	glcm	Idn
wavelet-HHH	glcm	Idm
wavelet-HHH	glcm	Correlation
wavelet-HHH	glcm	Autocorrelation
wavelet-HHH	glcm	SumEntropy
wavelet-HHH	glcm	SumSquares
wavelet-HHH	glcm	ClusterProminence
wavelet-HHH	glcm	Imc2
wavelet-HHH	glcm	Imc1
wavelet-HHH	glcm	DifferenceAverage

wavelet-HHH	glcm	Id
wavelet-HHH	glcm	ClusterTendency
wavelet-HHH	firstorder	InterquartileRange
wavelet-HHH	firstorder	Skewness
wavelet-HHH	firstorder	Uniformity
wavelet-HHH	firstorder	Median
wavelet-HHH	firstorder	Energy
wavelet-HHH	firstorder	RobustMeanAbsoluteDeviation
wavelet-HHH	firstorder	MeanAbsoluteDeviation
wavelet-HHH	firstorder	TotalEnergy
wavelet-HHH	firstorder	Maximum
wavelet-HHH	firstorder	RootMeanSquared
wavelet-HHH	firstorder	90Percentile
wavelet-HHH	firstorder	Minimum
wavelet-HHH	firstorder	Entropy
wavelet-HHH	firstorder	Range
wavelet-HHH	firstorder	Variance
wavelet-HHH	firstorder	10Percentile
wavelet-HHH	firstorder	Kurtosis
wavelet-HHH	firstorder	Mean
wavelet-HHH	glrlm	ShortRunLowGrayLevelEmphasis
wavelet-HHH	glrlm	GrayLevelVariance
wavelet-HHH	glrlm	LowGrayLevelRunEmphasis
wavelet-HHH	glrlm	GrayLevelNonUniformityNormalized
wavelet-HHH	glrlm	RunVariance
wavelet-HHH	glrlm	GrayLevelNonUniformity
wavelet-HHH	glrlm	LongRunEmphasis
wavelet-HHH	glrlm	ShortRunHighGrayLevelEmphasis
wavelet-HHH	glrlm	RunLengthNonUniformity
wavelet-HHH	glrlm	ShortRunEmphasis
wavelet-HHH	glrlm	LongRunHighGrayLevelEmphasis
wavelet-HHH	glrlm	RunPercentage
wavelet-HHH	glrlm	LongRunLowGrayLevelEmphasis
wavelet-HHH	glrlm	RunEntropy
wavelet-HHH	glrlm	HighGrayLevelRunEmphasis
wavelet-HHH	glrlm	RunLengthNonUniformityNormalized
wavelet-HHH	glszm	GrayLevelVariance
wavelet-HHH	glszm	ZoneVariance
wavelet-HHH	glszm	GrayLevelNonUniformityNormalized
wavelet-HHH	glszm	SizeZoneNonUniformityNormalized
wavelet-HHH	glszm	SizeZoneNonUniformity
wavelet-HHH	glszm	GrayLevelNonUniformity
wavelet-HHH	glszm	LargeAreaEmphasis
wavelet-HHH	glszm	SmallAreaHighGrayLevelEmphasis
wavelet-HHH	glszm	ZonePercentage
wavelet-HHH	glszm	LargeAreaLowGrayLevelEmphasis
wavelet-HHH	glszm	LargeAreaHighGrayLevelEmphasis

wavelet-HHH	glszm	HighGrayLevelZoneEmphasis
wavelet-HHH	glszm	SmallAreaEmphasis
wavelet-HHH	glszm	LowGrayLevelZoneEmphasis
wavelet-HHH	glszm	ZoneEntropy
wavelet-HHH	glszm	SmallAreaLowGrayLevelEmphasis
wavelet-HHH	ngtdm	Coarseness
wavelet-HHH	ngtdm	Complexity
wavelet-HHH	ngtdm	Strength
wavelet-HHH	ngtdm	Contrast
wavelet-HHH	ngtdm	Busyness
wavelet-HLL	gldm	GrayLevelVariance
wavelet-HLL	gldm	HighGrayLevelEmphasis
wavelet-HLL	gldm	DependenceEntropy
wavelet-HLL	gldm	DependenceNonUniformity
wavelet-HLL	gldm	GrayLevelNonUniformity
wavelet-HLL	gldm	SmallDependenceEmphasis
wavelet-HLL	gldm	SmallDependenceHighGrayLevelEmphasis
wavelet-HLL	gldm	DependenceNonUniformityNormalized
wavelet-HLL	gldm	LargeDependenceEmphasis
wavelet-HLL	gldm	LargeDependenceLowGrayLevelEmphasis
wavelet-HLL	gldm	DependenceVariance
wavelet-HLL	gldm	LargeDependenceHighGrayLevelEmphasis
wavelet-HLL	gldm	SmallDependenceLowGrayLevelEmphasis
wavelet-HLL	gldm	LowGrayLevelEmphasis
wavelet-HLL	glcm	JointAverage
wavelet-HLL	glcm	SumAverage
wavelet-HLL	glcm	JointEntropy
wavelet-HLL	glcm	ClusterShade
wavelet-HLL	glcm	MaximumProbability
wavelet-HLL	glcm	Idmn
wavelet-HLL	glcm	JointEnergy
wavelet-HLL	glcm	Contrast
wavelet-HLL	glcm	DifferenceEntropy
wavelet-HLL	glcm	InverseVariance
wavelet-HLL	glcm	DifferenceVariance
wavelet-HLL	glcm	Idn
wavelet-HLL	glcm	Idm
wavelet-HLL	glcm	Correlation
wavelet-HLL	glcm	Autocorrelation
wavelet-HLL	glcm	SumEntropy
wavelet-HLL	glcm	SumSquares
wavelet-HLL	glcm	ClusterProminence
wavelet-HLL	glcm	Imc2
wavelet-HLL	glcm	Imc1
wavelet-HLL	glcm	DifferenceAverage
wavelet-HLL	glcm	Id
wavelet-HLL	glcm	ClusterTendency

wavelet-HLL	firstorder	InterquartileRange
wavelet-HLL	firstorder	Skewness
wavelet-HLL	firstorder	Uniformity
wavelet-HLL	firstorder	Median
wavelet-HLL	firstorder	Energy
wavelet-HLL	firstorder	RobustMeanAbsoluteDeviation
wavelet-HLL	firstorder	MeanAbsoluteDeviation
wavelet-HLL	firstorder	TotalEnergy
wavelet-HLL	firstorder	Maximum
wavelet-HLL	firstorder	RootMeanSquared
wavelet-HLL	firstorder	90Percentile
wavelet-HLL	firstorder	Minimum
wavelet-HLL	firstorder	Entropy
wavelet-HLL	firstorder	Range
wavelet-HLL	firstorder	Variance
wavelet-HLL	firstorder	10Percentile
wavelet-HLL	firstorder	Kurtosis
wavelet-HLL	firstorder	Mean
wavelet-HLL	glrlm	ShortRunLowGrayLevelEmphasis
wavelet-HLL	glrlm	GrayLevelVariance
wavelet-HLL	glrlm	LowGrayLevelRunEmphasis
wavelet-HLL	glrlm	GrayLevelNonUniformityNormalized
wavelet-HLL	glrlm	RunVariance
wavelet-HLL	glrlm	GrayLevelNonUniformity
wavelet-HLL	glrlm	LongRunEmphasis
wavelet-HLL	glrlm	ShortRunHighGrayLevelEmphasis
wavelet-HLL	glrlm	RunLengthNonUniformity
wavelet-HLL	glrlm	ShortRunEmphasis
wavelet-HLL	glrlm	LongRunHighGrayLevelEmphasis
wavelet-HLL	glrlm	RunPercentage
wavelet-HLL	glrlm	LongRunLowGrayLevelEmphasis
wavelet-HLL	glrlm	RunEntropy
wavelet-HLL	glrlm	HighGrayLevelRunEmphasis
wavelet-HLL	glrlm	RunLengthNonUniformityNormalized
wavelet-HLL	glszm	GrayLevelVariance
wavelet-HLL	glszm	ZoneVariance
wavelet-HLL	glszm	GrayLevelNonUniformityNormalized
wavelet-HLL	glszm	SizeZoneNonUniformityNormalized
wavelet-HLL	glszm	SizeZoneNonUniformity
wavelet-HLL	glszm	GrayLevelNonUniformity
wavelet-HLL	glszm	LargeAreaEmphasis
wavelet-HLL	glszm	SmallAreaHighGrayLevelEmphasis
wavelet-HLL	glszm	ZonePercentage
wavelet-HLL	glszm	LargeAreaLowGrayLevelEmphasis
wavelet-HLL	glszm	LargeAreaHighGrayLevelEmphasis
wavelet-HLL	glszm	HighGrayLevelZoneEmphasis
wavelet-HLL	glszm	SmallAreaEmphasis

wavelet-HLL	glszm	LowGrayLevelZoneEmphasis
wavelet-HLL	glszm	ZoneEntropy
wavelet-HLL	glszm	SmallAreaLowGrayLevelEmphasis
wavelet-HLL	ngtdm	Coarseness
wavelet-HLL	ngtdm	Complexity
wavelet-HLL	ngtdm	Strength
wavelet-HLL	ngtdm	Contrast
wavelet-HLL	ngtdm	Busyness
wavelet-LHL	gldm	GrayLevelVariance
wavelet-LHL	gldm	HighGrayLevelEmphasis
wavelet-LHL	gldm	DependenceEntropy
wavelet-LHL	gldm	DependenceNonUniformity
wavelet-LHL	gldm	GrayLevelNonUniformity
wavelet-LHL	gldm	SmallDependenceEmphasis
wavelet-LHL	gldm	SmallDependenceHighGrayLevelEmphasis
wavelet-LHL	gldm	DependenceNonUniformityNormalized
wavelet-LHL	gldm	LargeDependenceEmphasis
wavelet-LHL	gldm	LargeDependenceLowGrayLevelEmphasis
wavelet-LHL	gldm	DependenceVariance
wavelet-LHL	gldm	LargeDependenceHighGrayLevelEmphasis
wavelet-LHL	gldm	SmallDependenceLowGrayLevelEmphasis
wavelet-LHL	gldm	LowGrayLevelEmphasis
wavelet-LHL	glcm	JointAverage
wavelet-LHL	glcm	SumAverage
wavelet-LHL	glcm	JointEntropy
wavelet-LHL	glcm	ClusterShade
wavelet-LHL	glcm	MaximumProbability
wavelet-LHL	glcm	Idmn
wavelet-LHL	glcm	JointEnergy
wavelet-LHL	glcm	Contrast
wavelet-LHL	glcm	DifferenceEntropy
wavelet-LHL	glcm	InverseVariance
wavelet-LHL	glcm	DifferenceVariance
wavelet-LHL	glcm	Idn
wavelet-LHL	glcm	Idm
wavelet-LHL	glcm	Correlation
wavelet-LHL	glcm	Autocorrelation
wavelet-LHL	glcm	SumEntropy
wavelet-LHL	glcm	SumSquares
wavelet-LHL	glcm	ClusterProminence
wavelet-LHL	glcm	Imc2
wavelet-LHL	glcm	Imc1
wavelet-LHL	glcm	DifferenceAverage
wavelet-LHL	glcm	Id
wavelet-LHL	glcm	ClusterTendency
wavelet-LHL	firstorder	InterquartileRange
wavelet-LHL	firstorder	Skewness

wavelet-LHL	firstorder	Uniformity
wavelet-LHL	firstorder	Median
wavelet-LHL	firstorder	Energy
wavelet-LHL	firstorder	RobustMeanAbsoluteDeviation
wavelet-LHL	firstorder	MeanAbsoluteDeviation
wavelet-LHL	firstorder	TotalEnergy
wavelet-LHL	firstorder	Maximum
wavelet-LHL	firstorder	RootMeanSquared
wavelet-LHL	firstorder	90Percentile
wavelet-LHL	firstorder	Minimum
wavelet-LHL	firstorder	Entropy
wavelet-LHL	firstorder	Range
wavelet-LHL	firstorder	Variance
wavelet-LHL	firstorder	10Percentile
wavelet-LHL	firstorder	Kurtosis
wavelet-LHL	firstorder	Mean
wavelet-LHL	glrlm	ShortRunLowGrayLevelEmphasis
wavelet-LHL	glrlm	GrayLevelVariance
wavelet-LHL	glrlm	LowGrayLevelRunEmphasis
wavelet-LHL	glrlm	GrayLevelNonUniformityNormalized
wavelet-LHL	glrlm	RunVariance
wavelet-LHL	glrlm	GrayLevelNonUniformity
wavelet-LHL	glrlm	LongRunEmphasis
wavelet-LHL	glrlm	ShortRunHighGrayLevelEmphasis
wavelet-LHL	glrlm	RunLengthNonUniformity
wavelet-LHL	glrlm	ShortRunEmphasis
wavelet-LHL	glrlm	LongRunHighGrayLevelEmphasis
wavelet-LHL	glrlm	RunPercentage
wavelet-LHL	glrlm	LongRunLowGrayLevelEmphasis
wavelet-LHL	glrlm	RunEntropy
wavelet-LHL	glrlm	HighGrayLevelRunEmphasis
wavelet-LHL	glrlm	RunLengthNonUniformityNormalized
wavelet-LHL	glszm	GrayLevelVariance
wavelet-LHL	glszm	ZoneVariance
wavelet-LHL	glszm	GrayLevelNonUniformityNormalized
wavelet-LHL	glszm	SizeZoneNonUniformityNormalized
wavelet-LHL	glszm	SizeZoneNonUniformity
wavelet-LHL	glszm	GrayLevelNonUniformity
wavelet-LHL	glszm	LargeAreaEmphasis
wavelet-LHL	glszm	SmallAreaHighGrayLevelEmphasis
wavelet-LHL	glszm	ZonePercentage
wavelet-LHL	glszm	LargeAreaLowGrayLevelEmphasis
wavelet-LHL	glszm	LargeAreaHighGrayLevelEmphasis
wavelet-LHL	glszm	HighGrayLevelZoneEmphasis
wavelet-LHL	glszm	SmallAreaEmphasis
wavelet-LHL	glszm	LowGrayLevelZoneEmphasis
wavelet-LHL	glszm	ZoneEntropy

wavelet-LHL	glszm	SmallAreaLowGrayLevelEmphasis
wavelet-LHL	ngtdm	Coarseness
wavelet-LHL	ngtdm	Complexity
wavelet-LHL	ngtdm	Strength
wavelet-LHL	ngtdm	Contrast
wavelet-LHL	ngtdm	Busyness
wavelet-LHH	gldm	GrayLevelVariance
wavelet-LHH	gldm	HighGrayLevelEmphasis
wavelet-LHH	gldm	DependenceEntropy
wavelet-LHH	gldm	DependenceNonUniformity
wavelet-LHH	gldm	GrayLevelNonUniformity
wavelet-LHH	gldm	SmallDependenceEmphasis
wavelet-LHH	gldm	SmallDependenceHighGrayLevelEmphasis
wavelet-LHH	gldm	DependenceNonUniformityNormalized
wavelet-LHH	gldm	LargeDependenceEmphasis
wavelet-LHH	gldm	LargeDependenceLowGrayLevelEmphasis
wavelet-LHH	gldm	DependenceVariance
wavelet-LHH	gldm	LargeDependenceHighGrayLevelEmphasis
wavelet-LHH	gldm	SmallDependenceLowGrayLevelEmphasis
wavelet-LHH	gldm	LowGrayLevelEmphasis
wavelet-LHH	glcm	JointAverage
wavelet-LHH	glcm	SumAverage
wavelet-LHH	glcm	JointEntropy
wavelet-LHH	glcm	ClusterShade
wavelet-LHH	glcm	MaximumProbability
wavelet-LHH	glcm	Idmn
wavelet-LHH	glcm	JointEnergy
wavelet-LHH	glcm	Contrast
wavelet-LHH	glcm	DifferenceEntropy
wavelet-LHH	glcm	InverseVariance
wavelet-LHH	glcm	DifferenceVariance
wavelet-LHH	glcm	Idn
wavelet-LHH	glcm	Idm
wavelet-LHH	glcm	Correlation
wavelet-LHH	glcm	Autocorrelation
wavelet-LHH	glcm	SumEntropy
wavelet-LHH	glcm	SumSquares
wavelet-LHH	glcm	ClusterProminence
wavelet-LHH	glcm	Imc2
wavelet-LHH	glcm	Imc1
wavelet-LHH	glcm	DifferenceAverage
wavelet-LHH	glcm	Id
wavelet-LHH	glcm	ClusterTendency
wavelet-LHH	firstorder	InterquartileRange
wavelet-LHH	firstorder	Skewness
wavelet-LHH	firstorder	Uniformity
wavelet-LHH	firstorder	Median

wavelet-LHH	firstorder	Energy
wavelet-LHH	firstorder	RobustMeanAbsoluteDeviation
wavelet-LHH	firstorder	MeanAbsoluteDeviation
wavelet-LHH	firstorder	TotalEnergy
wavelet-LHH	firstorder	Maximum
wavelet-LHH	firstorder	RootMeanSquared
wavelet-LHH	firstorder	90Percentile
wavelet-LHH	firstorder	Minimum
wavelet-LHH	firstorder	Entropy
wavelet-LHH	firstorder	Range
wavelet-LHH	firstorder	Variance
wavelet-LHH	firstorder	10Percentile
wavelet-LHH	firstorder	Kurtosis
wavelet-LHH	firstorder	Mean
wavelet-LHH	glrlm	ShortRunLowGrayLevelEmphasis
wavelet-LHH	glrlm	GrayLevelVariance
wavelet-LHH	glrlm	LowGrayLevelRunEmphasis
wavelet-LHH	glrlm	GrayLevelNonUniformityNormalized
wavelet-LHH	glrlm	RunVariance
wavelet-LHH	glrlm	GrayLevelNonUniformity
wavelet-LHH	glrlm	LongRunEmphasis
wavelet-LHH	glrlm	ShortRunHighGrayLevelEmphasis
wavelet-LHH	glrlm	RunLengthNonUniformity
wavelet-LHH	glrlm	ShortRunEmphasis
wavelet-LHH	glrlm	LongRunHighGrayLevelEmphasis
wavelet-LHH	glrlm	RunPercentage
wavelet-LHH	glrlm	LongRunLowGrayLevelEmphasis
wavelet-LHH	glrlm	RunEntropy
wavelet-LHH	glrlm	HighGrayLevelRunEmphasis
wavelet-LHH	glrlm	RunLengthNonUniformityNormalized
wavelet-LHH	glszm	GrayLevelVariance
wavelet-LHH	glszm	ZoneVariance
wavelet-LHH	glszm	GrayLevelNonUniformityNormalized
wavelet-LHH	glszm	SizeZoneNonUniformityNormalized
wavelet-LHH	glszm	SizeZoneNonUniformity
wavelet-LHH	glszm	GrayLevelNonUniformity
wavelet-LHH	glszm	LargeAreaEmphasis
wavelet-LHH	glszm	SmallAreaHighGrayLevelEmphasis
wavelet-LHH	glszm	ZonePercentage
wavelet-LHH	glszm	LargeAreaLowGrayLevelEmphasis
wavelet-LHH	glszm	LargeAreaHighGrayLevelEmphasis
wavelet-LHH	glszm	HighGrayLevelZoneEmphasis
wavelet-LHH	glszm	SmallAreaEmphasis
wavelet-LHH	glszm	LowGrayLevelZoneEmphasis
wavelet-LHH	glszm	ZoneEntropy
wavelet-LHH	glszm	SmallAreaLowGrayLevelEmphasis
wavelet-LHH	ngtdm	Coarseness

wavelet-LHH	ngtdm	Complexity
wavelet-LHH	ngtdm	Strength
wavelet-LHH	ngtdm	Contrast
wavelet-LHH	ngtdm	Busyness
wavelet-LLH	gldm	GrayLevelVariance
wavelet-LLH	gldm	HighGrayLevelEmphasis
wavelet-LLH	gldm	DependenceEntropy
wavelet-LLH	gldm	DependenceNonUniformity
wavelet-LLH	gldm	GrayLevelNonUniformity
wavelet-LLH	gldm	SmallDependenceEmphasis
wavelet-LLH	gldm	SmallDependenceHighGrayLevelEmphasis
wavelet-LLH	gldm	DependenceNonUniformityNormalized
wavelet-LLH	gldm	LargeDependenceEmphasis
wavelet-LLH	gldm	LargeDependenceLowGrayLevelEmphasis
wavelet-LLH	gldm	DependenceVariance
wavelet-LLH	gldm	LargeDependenceHighGrayLevelEmphasis
wavelet-LLH	gldm	SmallDependenceLowGrayLevelEmphasis
wavelet-LLH	gldm	LowGrayLevelEmphasis
wavelet-LLH	glcm	JointAverage
wavelet-LLH	glcm	SumAverage
wavelet-LLH	glcm	JointEntropy
wavelet-LLH	glcm	ClusterShade
wavelet-LLH	glcm	MaximumProbability
wavelet-LLH	glcm	Idmn
wavelet-LLH	glcm	JointEnergy
wavelet-LLH	glcm	Contrast
wavelet-LLH	glcm	DifferenceEntropy
wavelet-LLH	glcm	InverseVariance
wavelet-LLH	glcm	DifferenceVariance
wavelet-LLH	glcm	Idn
wavelet-LLH	glcm	Idm
wavelet-LLH	glcm	Correlation
wavelet-LLH	glcm	Autocorrelation
wavelet-LLH	glcm	SumEntropy
wavelet-LLH	glcm	SumSquares
wavelet-LLH	glcm	ClusterProminence
wavelet-LLH	glcm	Imc2
wavelet-LLH	glcm	Imc1
wavelet-LLH	glcm	DifferenceAverage
wavelet-LLH	glcm	Id
wavelet-LLH	glcm	ClusterTendency
wavelet-LLH	firstorder	InterquartileRange
wavelet-LLH	firstorder	Skewness
wavelet-LLH	firstorder	Uniformity
wavelet-LLH	firstorder	Median
wavelet-LLH	firstorder	Energy
wavelet-LLH	firstorder	RobustMeanAbsoluteDeviation

wavelet-LLH	firstorder	MeanAbsoluteDeviation
wavelet-LLH	firstorder	TotalEnergy
wavelet-LLH	firstorder	Maximum
wavelet-LLH	firstorder	RootMeanSquared
wavelet-LLH	firstorder	90Percentile
wavelet-LLH	firstorder	Minimum
wavelet-LLH	firstorder	Entropy
wavelet-LLH	firstorder	Range
wavelet-LLH	firstorder	Variance
wavelet-LLH	firstorder	10Percentile
wavelet-LLH	firstorder	Kurtosis
wavelet-LLH	firstorder	Mean
wavelet-LLH	glrlm	ShortRunLowGrayLevelEmphasis
wavelet-LLH	glrlm	GrayLevelVariance
wavelet-LLH	glrlm	LowGrayLevelRunEmphasis
wavelet-LLH	glrlm	GrayLevelNonUniformityNormalized
wavelet-LLH	glrlm	RunVariance
wavelet-LLH	glrlm	GrayLevelNonUniformity
wavelet-LLH	glrlm	LongRunEmphasis
wavelet-LLH	glrlm	ShortRunHighGrayLevelEmphasis
wavelet-LLH	glrlm	RunLengthNonUniformity
wavelet-LLH	glrlm	ShortRunEmphasis
wavelet-LLH	glrlm	LongRunHighGrayLevelEmphasis
wavelet-LLH	glrlm	RunPercentage
wavelet-LLH	glrlm	LongRunLowGrayLevelEmphasis
wavelet-LLH	glrlm	RunEntropy
wavelet-LLH	glrlm	HighGrayLevelRunEmphasis
wavelet-LLH	glrlm	RunLengthNonUniformityNormalized
wavelet-LLH	glszm	GrayLevelVariance
wavelet-LLH	glszm	ZoneVariance
wavelet-LLH	glszm	GrayLevelNonUniformityNormalized
wavelet-LLH	glszm	SizeZoneNonUniformityNormalized
wavelet-LLH	glszm	SizeZoneNonUniformity
wavelet-LLH	glszm	GrayLevelNonUniformity
wavelet-LLH	glszm	LargeAreaEmphasis
wavelet-LLH	glszm	SmallAreaHighGrayLevelEmphasis
wavelet-LLH	glszm	ZonePercentage
wavelet-LLH	glszm	LargeAreaLowGrayLevelEmphasis
wavelet-LLH	glszm	LargeAreaHighGrayLevelEmphasis
wavelet-LLH	glszm	HighGrayLevelZoneEmphasis
wavelet-LLH	glszm	SmallAreaEmphasis
wavelet-LLH	glszm	LowGrayLevelZoneEmphasis
wavelet-LLH	glszm	ZoneEntropy
wavelet-LLH	glszm	SmallAreaLowGrayLevelEmphasis
wavelet-LLH	ngtdm	Coarseness
wavelet-LLH	ngtdm	Complexity
wavelet-LLH	ngtdm	Strength

wavelet-LLH	ngtdm	Contrast
wavelet-LLH	ngtdm	Busyness
wavelet-LLL	gldm	GrayLevelVariance
wavelet-LLL	gldm	HighGrayLevelEmphasis
wavelet-LLL	gldm	DependenceEntropy
wavelet-LLL	gldm	DependenceNonUniformity
wavelet-LLL	gldm	GrayLevelNonUniformity
wavelet-LLL	gldm	SmallDependenceEmphasis
wavelet-LLL	gldm	SmallDependenceHighGrayLevelEmphasis
wavelet-LLL	gldm	DependenceNonUniformityNormalized
wavelet-LLL	gldm	LargeDependenceEmphasis
wavelet-LLL	gldm	LargeDependenceLowGrayLevelEmphasis
wavelet-LLL	gldm	DependenceVariance
wavelet-LLL	gldm	LargeDependenceHighGrayLevelEmphasis
wavelet-LLL	gldm	SmallDependenceLowGrayLevelEmphasis
wavelet-LLL	gldm	LowGrayLevelEmphasis
wavelet-LLL	glcm	JointAverage
wavelet-LLL	glcm	SumAverage
wavelet-LLL	glcm	JointEntropy
wavelet-LLL	glcm	ClusterShade
wavelet-LLL	glcm	MaximumProbability
wavelet-LLL	glcm	Idmn
wavelet-LLL	glcm	JointEnergy
wavelet-LLL	glcm	Contrast
wavelet-LLL	glcm	DifferenceEntropy
wavelet-LLL	glcm	InverseVariance
wavelet-LLL	glcm	DifferenceVariance
wavelet-LLL	glcm	Idn
wavelet-LLL	glcm	Idm
wavelet-LLL	glcm	Correlation
wavelet-LLL	glcm	Autocorrelation
wavelet-LLL	glcm	SumEntropy
wavelet-LLL	glcm	SumSquares
wavelet-LLL	glcm	ClusterProminence
wavelet-LLL	glcm	Imc2
wavelet-LLL	glcm	Imc1
wavelet-LLL	glcm	DifferenceAverage
wavelet-LLL	glcm	Id
wavelet-LLL	glcm	ClusterTendency
wavelet-LLL	firstorder	InterquartileRange
wavelet-LLL	firstorder	Skewness
wavelet-LLL	firstorder	Uniformity
wavelet-LLL	firstorder	Median
wavelet-LLL	firstorder	Energy
wavelet-LLL	firstorder	RobustMeanAbsoluteDeviation
wavelet-LLL	firstorder	MeanAbsoluteDeviation
wavelet-LLL	firstorder	TotalEnergy

wavelet-LLL	firstorder	Maximum
wavelet-LLL	firstorder	RootMeanSquared
wavelet-LLL	firstorder	90Percentile
wavelet-LLL	firstorder	Minimum
wavelet-LLL	firstorder	Entropy
wavelet-LLL	firstorder	Range
wavelet-LLL	firstorder	Variance
wavelet-LLL	firstorder	10Percentile
wavelet-LLL	firstorder	Kurtosis
wavelet-LLL	firstorder	Mean
wavelet-LLL	glrlm	ShortRunLowGrayLevelEmphasis
wavelet-LLL	glrlm	GrayLevelVariance
wavelet-LLL	glrlm	LowGrayLevelRunEmphasis
wavelet-LLL	glrlm	GrayLevelNonUniformityNormalized
wavelet-LLL	glrlm	RunVariance
wavelet-LLL	glrlm	GrayLevelNonUniformity
wavelet-LLL	glrlm	LongRunEmphasis
wavelet-LLL	glrlm	ShortRunHighGrayLevelEmphasis
wavelet-LLL	glrlm	RunLengthNonUniformity
wavelet-LLL	glrlm	ShortRunEmphasis
wavelet-LLL	glrlm	LongRunHighGrayLevelEmphasis
wavelet-LLL	glrlm	RunPercentage
wavelet-LLL	glrlm	LongRunLowGrayLevelEmphasis
wavelet-LLL	glrlm	RunEntropy
wavelet-LLL	glrlm	HighGrayLevelRunEmphasis
wavelet-LLL	glrlm	RunLengthNonUniformityNormalized
wavelet-LLL	glszm	GrayLevelVariance
wavelet-LLL	glszm	ZoneVariance
wavelet-LLL	glszm	GrayLevelNonUniformityNormalized
wavelet-LLL	glszm	SizeZoneNonUniformityNormalized
wavelet-LLL	glszm	SizeZoneNonUniformity
wavelet-LLL	glszm	GrayLevelNonUniformity
wavelet-LLL	glszm	LargeAreaEmphasis
wavelet-LLL	glszm	SmallAreaHighGrayLevelEmphasis
wavelet-LLL	glszm	ZonePercentage
wavelet-LLL	glszm	LargeAreaLowGrayLevelEmphasis
wavelet-LLL	glszm	LargeAreaHighGrayLevelEmphasis
wavelet-LLL	glszm	HighGrayLevelZoneEmphasis
wavelet-LLL	glszm	SmallAreaEmphasis
wavelet-LLL	glszm	LowGrayLevelZoneEmphasis
wavelet-LLL	glszm	ZoneEntropy
wavelet-LLL	glszm	SmallAreaLowGrayLevelEmphasis
wavelet-LLL	ngtdm	Coarseness
wavelet-LLL	ngtdm	Complexity
wavelet-LLL	ngtdm	Strength
wavelet-LLL	ngtdm	Contrast
wavelet-LLL	ngtdm	Busyness

wavelet-HHL	gldm	GrayLevelVariance
wavelet-HHL	gldm	HighGrayLevelEmphasis
wavelet-HHL	gldm	DependenceEntropy
wavelet-HHL	gldm	DependenceNonUniformity
wavelet-HHL	gldm	GrayLevelNonUniformity
wavelet-HHL	gldm	SmallDependenceEmphasis
wavelet-HHL	gldm	SmallDependenceHighGrayLevelEmphasis
wavelet-HHL	gldm	DependenceNonUniformityNormalized
wavelet-HHL	gldm	LargeDependenceEmphasis
wavelet-HHL	gldm	LargeDependenceLowGrayLevelEmphasis
wavelet-HHL	gldm	DependenceVariance
wavelet-HHL	gldm	LargeDependenceHighGrayLevelEmphasis
wavelet-HHL	gldm	SmallDependenceLowGrayLevelEmphasis
wavelet-HHL	gldm	LowGrayLevelEmphasis
wavelet-HHL	glcm	JointAverage
wavelet-HHL	glcm	SumAverage
wavelet-HHL	glcm	JointEntropy
wavelet-HHL	glcm	ClusterShade
wavelet-HHL	glcm	MaximumProbability
wavelet-HHL	glcm	Idmn
wavelet-HHL	glcm	JointEnergy
wavelet-HHL	glcm	Contrast
wavelet-HHL	glcm	DifferenceEntropy
wavelet-HHL	glcm	InverseVariance
wavelet-HHL	glcm	DifferenceVariance
wavelet-HHL	glcm	Idn
wavelet-HHL	glcm	Idm
wavelet-HHL	glcm	Correlation
wavelet-HHL	glcm	Autocorrelation
wavelet-HHL	glcm	SumEntropy
wavelet-HHL	glcm	SumSquares
wavelet-HHL	glcm	ClusterProminence
wavelet-HHL	glcm	Imc2
wavelet-HHL	glcm	Imc1
wavelet-HHL	glcm	DifferenceAverage
wavelet-HHL	glcm	Id
wavelet-HHL	glcm	ClusterTendency
wavelet-HHL	firstorder	InterquartileRange
wavelet-HHL	firstorder	Skewness
wavelet-HHL	firstorder	Uniformity
wavelet-HHL	firstorder	Median
wavelet-HHL	firstorder	Energy
wavelet-HHL	firstorder	RobustMeanAbsoluteDeviation
wavelet-HHL	firstorder	MeanAbsoluteDeviation
wavelet-HHL	firstorder	TotalEnergy
wavelet-HHL	firstorder	Maximum
wavelet-HHL	firstorder	RootMeanSquared

wavelet-HHL	firstorder	90Percentile
wavelet-HHL	firstorder	Minimum
wavelet-HHL	firstorder	Entropy
wavelet-HHL	firstorder	Range
wavelet-HHL	firstorder	Variance
wavelet-HHL	firstorder	10Percentile
wavelet-HHL	firstorder	Kurtosis
wavelet-HHL	firstorder	Mean
wavelet-HHL	glrlm	ShortRunLowGrayLevelEmphasis
wavelet-HHL	glrlm	GrayLevelVariance
wavelet-HHL	glrlm	LowGrayLevelRunEmphasis
wavelet-HHL	glrlm	GrayLevelNonUniformityNormalized
wavelet-HHL	glrlm	RunVariance
wavelet-HHL	glrlm	GrayLevelNonUniformity
wavelet-HHL	glrlm	LongRunEmphasis
wavelet-HHL	glrlm	ShortRunHighGrayLevelEmphasis
wavelet-HHL	glrlm	RunLengthNonUniformity
wavelet-HHL	glrlm	ShortRunEmphasis
wavelet-HHL	glrlm	LongRunHighGrayLevelEmphasis
wavelet-HHL	glrlm	RunPercentage
wavelet-HHL	glrlm	LongRunLowGrayLevelEmphasis
wavelet-HHL	glrlm	RunEntropy
wavelet-HHL	glrlm	HighGrayLevelRunEmphasis
wavelet-HHL	glrlm	RunLengthNonUniformityNormalized
wavelet-HHL	glszm	GrayLevelVariance
wavelet-HHL	glszm	ZoneVariance
wavelet-HHL	glszm	GrayLevelNonUniformityNormalized
wavelet-HHL	glszm	SizeZoneNonUniformityNormalized
wavelet-HHL	glszm	SizeZoneNonUniformity
wavelet-HHL	glszm	GrayLevelNonUniformity
wavelet-HHL	glszm	LargeAreaEmphasis
wavelet-HHL	glszm	SmallAreaHighGrayLevelEmphasis
wavelet-HHL	glszm	ZonePercentage
wavelet-HHL	glszm	LargeAreaLowGrayLevelEmphasis
wavelet-HHL	glszm	LargeAreaHighGrayLevelEmphasis
wavelet-HHL	glszm	HighGrayLevelZoneEmphasis
wavelet-HHL	glszm	SmallAreaEmphasis
wavelet-HHL	glszm	LowGrayLevelZoneEmphasis
wavelet-HHL	glszm	ZoneEntropy
wavelet-HHL	glszm	SmallAreaLowGrayLevelEmphasis
wavelet-HHL	ngtdm	Coarseness
wavelet-HHL	ngtdm	Complexity
wavelet-HHL	ngtdm	Strength
wavelet-HHL	ngtdm	Contrast
wavelet-HHL	ngtdm	Busyness
wavelet-HLH	gldm	GrayLevelVariance
wavelet-HLH	gldm	HighGrayLevelEmphasis

wavelet-HLH	gldm	DependenceEntropy
wavelet-HLH	gldm	DependenceNonUniformity
wavelet-HLH	gldm	GrayLevelNonUniformity
wavelet-HLH	gldm	SmallDependenceEmphasis
wavelet-HLH	gldm	SmallDependenceHighGrayLevelEmphasis
wavelet-HLH	gldm	DependenceNonUniformityNormalized
wavelet-HLH	gldm	LargeDependenceEmphasis
wavelet-HLH	gldm	LargeDependenceLowGrayLevelEmphasis
wavelet-HLH	gldm	DependenceVariance
wavelet-HLH	gldm	LargeDependenceHighGrayLevelEmphasis
wavelet-HLH	gldm	SmallDependenceLowGrayLevelEmphasis
wavelet-HLH	gldm	LowGrayLevelEmphasis
wavelet-HLH	glcm	JointAverage
wavelet-HLH	glcm	SumAverage
wavelet-HLH	glcm	JointEntropy
wavelet-HLH	glcm	ClusterShade
wavelet-HLH	glcm	MaximumProbability
wavelet-HLH	glcm	Idmn
wavelet-HLH	glcm	JointEnergy
wavelet-HLH	glcm	Contrast
wavelet-HLH	glcm	DifferenceEntropy
wavelet-HLH	glcm	InverseVariance
wavelet-HLH	glcm	DifferenceVariance
wavelet-HLH	glcm	Idn
wavelet-HLH	glcm	Idm
wavelet-HLH	glcm	Correlation
wavelet-HLH	glcm	Autocorrelation
wavelet-HLH	glcm	SumEntropy
wavelet-HLH	glcm	SumSquares
wavelet-HLH	glcm	ClusterProminence
wavelet-HLH	glcm	Imc2
wavelet-HLH	glcm	Imc1
wavelet-HLH	glcm	DifferenceAverage
wavelet-HLH	glcm	Id
wavelet-HLH	glcm	ClusterTendency
wavelet-HLH	firstorder	InterquartileRange
wavelet-HLH	firstorder	Skewness
wavelet-HLH	firstorder	Uniformity
wavelet-HLH	firstorder	Median
wavelet-HLH	firstorder	Energy
wavelet-HLH	firstorder	RobustMeanAbsoluteDeviation
wavelet-HLH	firstorder	MeanAbsoluteDeviation
wavelet-HLH	firstorder	TotalEnergy
wavelet-HLH	firstorder	Maximum
wavelet-HLH	firstorder	RootMeanSquared
wavelet-HLH	firstorder	90Percentile
wavelet-HLH	firstorder	Minimum

wavelet-HLH	firstorder	Entropy
wavelet-HLH	firstorder	Range
wavelet-HLH	firstorder	Variance
wavelet-HLH	firstorder	10Percentile
wavelet-HLH	firstorder	Kurtosis
wavelet-HLH	firstorder	Mean
wavelet-HLH	glrlm	ShortRunLowGrayLevelEmphasis
wavelet-HLH	glrlm	GrayLevelVariance
wavelet-HLH	glrlm	LowGrayLevelRunEmphasis
wavelet-HLH	glrlm	GrayLevelNonUniformityNormalized
wavelet-HLH	glrlm	RunVariance
wavelet-HLH	glrlm	GrayLevelNonUniformity
wavelet-HLH	glrlm	LongRunEmphasis
wavelet-HLH	glrlm	ShortRunHighGrayLevelEmphasis
wavelet-HLH	glrlm	RunLengthNonUniformity
wavelet-HLH	glrlm	ShortRunEmphasis
wavelet-HLH	glrlm	LongRunHighGrayLevelEmphasis
wavelet-HLH	glrlm	RunPercentage
wavelet-HLH	glrlm	LongRunLowGrayLevelEmphasis
wavelet-HLH	glrlm	RunEntropy
wavelet-HLH	glrlm	HighGrayLevelRunEmphasis
wavelet-HLH	glrlm	RunLengthNonUniformityNormalized
wavelet-HLH	glszm	GrayLevelVariance
wavelet-HLH	glszm	ZoneVariance
wavelet-HLH	glszm	GrayLevelNonUniformityNormalized
wavelet-HLH	glszm	SizeZoneNonUniformityNormalized
wavelet-HLH	glszm	SizeZoneNonUniformity
wavelet-HLH	glszm	GrayLevelNonUniformity
wavelet-HLH	glszm	LargeAreaEmphasis
wavelet-HLH	glszm	SmallAreaHighGrayLevelEmphasis
wavelet-HLH	glszm	ZonePercentage
wavelet-HLH	glszm	LargeAreaLowGrayLevelEmphasis
wavelet-HLH	glszm	LargeAreaHighGrayLevelEmphasis
wavelet-HLH	glszm	HighGrayLevelZoneEmphasis
wavelet-HLH	glszm	SmallAreaEmphasis
wavelet-HLH	glszm	LowGrayLevelZoneEmphasis
wavelet-HLH	glszm	ZoneEntropy
wavelet-HLH	glszm	SmallAreaLowGrayLevelEmphasis
wavelet-HLH	ngtdm	Coarseness
wavelet-HLH	ngtdm	Complexity
wavelet-HLH	ngtdm	Strength
wavelet-HLH	ngtdm	Contrast
wavelet-HLH	ngtdm	Busyness
